# Critical Insights into Parathyroid Cancer

**DOI:** 10.26502/jcsct.5079285

**Published:** 2026-06-24

**Authors:** Tariq Ali Masri-zada, Matteo Giovanni Candela, Hassan Makki, Hany Salman, Shadi Aziz, Nadine Bazzi, Devendra K. Agrawal

**Affiliations:** 1 Department of Translational Research, College of Osteopathic Medicine of the Pacific, Western University of Health Sciences, Pomona, CA 91766, USA; 2 Lake Erie College of Osteopathic Medicine, Erie, PA 16509, USA; 3 College of Osteopathic Medicine, William Carey University, Hattiesburg, MS 39401, USA; 4 School of Health Sciences, Oakland University, Rochester, MI 48309, USA; 5 Michigan State University College of Osteopathic Medicine, East Lansing, MI 48824, USA

**Keywords:** Cancer, Carcinoma, CDC73, En Bloc Resection, Endocrine malignancy, Hypercalcemia, Ki-67, Parafibromin, Parathyroid adenoma, Parathyroid carcinoma

## Abstract

Parathyroid carcinoma (PC) is a rare endocrine malignancy accounting for less than 1% of primary hyperparathyroidism cases. It carries significant morbidity due to severe hypercalcemia and a high rate of recurrence post-resection. This literature review synthesizes current evidence on the diagnostic challenges, surgical management, and outcomes associated with parathyroid carcinoma. Diagnosis of parathyroid carcinoma remains difficult due to a large overlap with parathyroid adenoma in clinical presentation, laboratory findings, and imaging characteristics. However, markedly elevated serum calcium and parathyroid hormone levels, palpable neck mass, and evidence of local invasion may raise suspicion for malignancy. Definitive diagnosis relies on histopathologic features such as capsular, vascular, or perineural invasion. Also, many immunohistochemical markers are currently under review, including mutation of the CDC73 gene, loss of parafibromin, and elevated Ki-67, which support the diagnosis. Complete *en bloc* resection at the initial operation represents the most critical determinant of long-term outcomes. Achieving negative margins significantly reduces recurrence rates and improves survival, while limited resection and capsular disruption are associated with increased risk of locoregional recurrence. While lymph node involvement is relatively uncommon, selective rather than routine lymph node dissection is recommended. Adjuvant therapies, including radiation and systemic treatments, play a limited and largely palliative role, particularly in advanced or unresectable disease. Parathyroid carcinoma commonly recurs, requiring multiple reoperations, with morbidity primarily driven by persistent hypercalcemia. Long-term surveillance with serial biochemical monitoring is essential due to the risk of late recurrence. Overall, evidence consistently demonstrates that outcomes are contingent on the adequacy of the initial surgical resection, highlighting the importance of early recognition and complete *en bloc* resection.

## 1. Introduction

Parathyroid carcinoma, which constitutes less than 1% of all cases of primary hyperparathyroidism, is a rare but important endocrine malignancy that arises from the parathyroid glands [1–4]. Although uncommon, parathyroid carcinoma can cause significant morbidity due to severe hypercalcemia, end organ damage, and a high risk of recurrence if not properly treated during the initial surgery [5–7]. As opposed to benign pathologies involving the parathyroid gland including parathyroid adenoma or hyperplasia, parathyroid carcinoma has a more aggressive clinical course, with invasion into surrounding tissues and structures, involvement of regional lymph nodes, and potential distant metastasis [7–9]. Early recognition is crucial, as complete surgical resection is the most important factor impacting long-term prognosis and disease control [9–12]. Recent literature emphasizes notably worse outcomes and increased recurrence rates in parathyroid carcinoma associated with insufficient or delayed surgical management [13]. PC most commonly presents between the fourth and sixth decades of life and affects men and women at similar rates, unlike benign primary hyperparathyroidism, which occurs more frequently in women [7, 14, 15]. Parathyroid hormone (PTH) is the primary cause of symptoms, as excessive release induces severe hypercalcemia and its systemic effects [7, 8]. Presenting features include nephrolithiasis, polyuria, neurocognitive symptoms, bone pain, gastrointestinal symptoms, and fatigue [7, 10]. Compared to benign hyperparathyroidism, the incidence of skeletal pathologies, including pathologic fractures and osteitis fibrosa cystica, is much higher in malignant disease, demonstrating the severity and prolonged elevations in PTH and subsequent calcium levels [8, 16].

**Figure 1:**
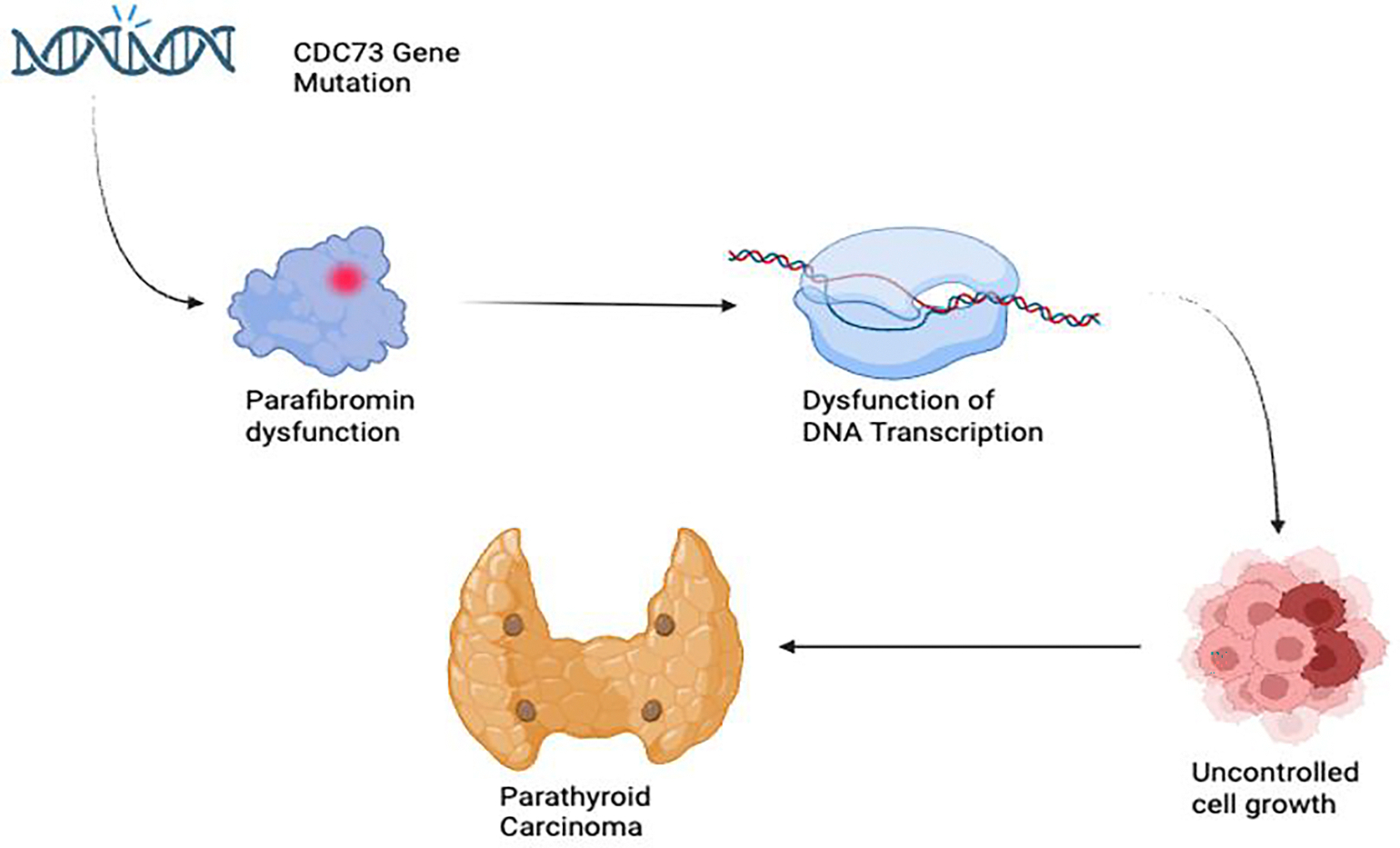
Genetic factor in the pathogenesis of parathyroid carcinoma.

Genetic factors also play an important role in the development of PC (Figure 1). The most prominent molecular abnormalities related to this condition are mutations in the CDC73 tumor suppressor gene [7, 17]. The CDC73 gene encodes parafibromin, a protein responsible for regulating transcription and cell growth. Uncontrolled cellular proliferation and malignant transformation can result from parafibromin dysfunction [7]. In addition to the markedly elevated risk of developing PC, germline mutations in CDC73 are also related to hyperparathyroidism-jaw tumor syndrome [7, 20]. Other genetic associations include multiple endocrine neoplasia syndromes, particularly MEN1 and MEN2; however, PC is rare in these conditions compared to benign disease [7, 22, 23]. Additionally, growing evidence suggests that genetic and molecular profiling may improve diagnostic accuracy and help guide management in PC [24] (Figure 1).

Parathyroid carcinoma is characterized by a high rate of recurrence, which can occur years after the initial surgical operation. Reported recurrence rates range from 30% to 60%, with persistent or recurrent hypercalcemia being the main cause of morbidity and mortality [7–11,14]. The most common sites of metastases include bone, lungs, liver, and regional lymph nodes [7, 9, 10]. Notably, mortality more often results from complications of uncontrolled hypercalcemia rather than tumor burden itself; therefore, early diagnosis and appropriate surgical management are essential [7, 18].

## 2. Diagnostic Challenges and Preoperative Evaluation

Preoperative diagnosis of parathyroid carcinoma continues to be difficult because its clinical presentation, laboratory findings, and imaging features often overlap with those of benign primary hyperparathyroidism [6, 7, 15]. In many cases, a definitive diagnosis is only made after surgical resection and histological evaluation. However, certain clinical and laboratory findings may suggest underlying malignancy [7,8,10] (Table 1). Patients with PC commonly present with significantly elevated PTH levels, often three to ten times higher than the upper limit of normal, as well as markedly elevated serum calcium levels, frequently greater than 14 mg/dL [7, 8, 10, 15]. Compared to the findings in benign hyperparathyroidism, these abnormalities are typically more severe [7]. The presence of a palpable neck mass is also notable, as benign parathyroid adenomas are rarely palpable [7, 14]. Complications associated with severe hypercalcemia, including neurocognitive symptoms, skeletal pathologies, renal insufficiency, and nephrolithiasis, may also be present [7, 10, 16].

Although imaging cannot reliably differentiate benign from malignant disease, it is crucial for localization and operative approach [6, 7]. The first imaging study performed is typically a neck ultrasound, which can identify enlarged glands and evaluate surrounding structures [6]. Findings suggestive of malignancy include heterogeneous echotexture, irregular borders, and evidence of invasion [6, 7]. Technetium-99m sestamibi scanning is widely used to localize hyperfunctioning parathyroid tissue; however, it cannot definitively differentiate adenomas from carcinomas [6]. CT and MRI cross-sectional imaging can provide additional information concerning tumor size, local and regional invasion, and metastatic disease [6, 9]. Fine-needle aspiration biopsy is typically avoided in cases where parathyroid carcinoma is suspected due to the risk of tumor seeding and low diagnostic yield [7, 19]. Cytologic findings alone cannot reliably distinguish carcinoma from adenoma, and definitive diagnosis requires histologic evidence of capsular or vascular invasion or metastatic disease [7, 20]. Preoperative suspicion is important because it affects the operative approach. At the initial surgical intervention, *en bloc* resection has been linked to improved long-term outcomes compared to limited excision [9–12].

## 3. Diagnosis and Distinguishing Parathyroid Carcinoma and Adenoma

PC and parathyroid adenomas can sometimes present similarly but have distinct clinical, immunohistochemical, and genomic features. Biochemically, malignant disease is associated with elevated lab levels such as serum calcium reported at 13.0 compared to 11.8 mg/dL, as well as iPTH at 489 compared to 266 pg/mL [25]. A retrospective study across 28 patients also found that profound hypercalcemia (≥14 mg/dL) was more common in PC in 62% of patients compared to atypical adenomas at 17% [26]. A definitive diagnosis of malignant growth requires the demonstration of invasive features such as angioinvasion, perineural invasion, or direct extension into adjacent tissues [27]. These findings are absent in adenomas and allow the clinician to distinguish parathyroid carcinoma from benign disease with greater diagnostic certainty, particularly in cases where preoperative and intraoperative findings are equivocal.

## 4. Histopathology and Biochemical Markers

Histopathological features of parathyroid carcinomas are also significant in the diagnosis of malignancy. Morphological features of the growths that signify parathyroid carcinoma rather than adenoma include a trabecular growth pattern, broad fibrous bands, and a higher mitotic index measured as >5 mitoses/50 high power field, atypical nuclei, and necrosis [28,29]. Another common feature that helps to differentiate the two is a lack of a distinct capsule and invasion within adjacent structures [25]. There are also immunohistochemical markers that may aid in distinguishing parathyroid carcinoma from adenoma, although none are independently diagnostic. Parafibromin, a tumor suppressor protein involved in transcriptional regulation and encoded by the CDC73 gene, has emerged as one of the most clinically relevant markers. Loss of parafibromin expression is highly specific for parathyroid carcinoma and has been associated with more aggressive disease behavior. It is also linked to hereditary conditions such as hyperparathyroidism-jaw tumor (HPT-JT) syndrome, as well as a subset of sporadic parathyroid neoplasms [30]. Loss of nuclear parafibromin expression has been reported in approximately 31–73% of carcinomas, compared to 0–1% in adenomas [31, 32, 33]. Importantly, normal parafibromin expression is not consistent with malignancy, with a negative predictive value of 90–98% in many cases [34]. Notably, all metastatic carcinomas in one large series demonstrated loss of parafibromin expression, whereas none of the adenomas did [35]. Another single marker that is significant in discriminating for malignancy is Ki-67. In a study looking at several cases of adenoma and carcinoma, the presence and absence of the marker was significantly elevated at 86% in the parathyroid carcinomas as opposed to 0% of adenomas [33]. There have been other studies, however, that report elevated Ki-67 in carcinoma but a mild presence in 0–5% of adenomas [31, 33]. The combination of Galectin-3 overexpression along with elevated Ki-67 expression is reported to be suggestive of carcinoma, as studied by Bergero and colleagues [36].

## 5. Features of Benign Growth

Parathyroid adenoma is the most common cause of primary hyperparathyroidism (PHPT). It ends up accounting for around 80–85% of cases, with multi gland hyperplasia and carcinoma comprising the remainder [38,40]. PHPT itself is relatively common, with a prevalence of approximately 0.1–0.4% in the general population, which increases with age. Data shows a female predominance (≈3–4:1) and most frequently presents in the 5th–7th decades of life [38, 39]. Unlike carcinoma, parathyroid adenomas are typically sporadic and benign, although they may occur in hereditary syndromes such as MEN1 or MEN2A [40, 41]. Clinically speaking, parathyroid adenoma normally presents as asymptomatic or mildly symptomatic hypercalcemia. It is frequently identified incidentally on routine laboratory testing in modern practice [38, 42]. When symptoms present, they tend to be subtle and chronic, including fatigue, mild cognitive changes, nephrolithiasis, and decreased bone density. This is in clear opposition to the severe skeletal and renal complications seen in carcinoma [39, 42]. Biochemically speaking, patients exhibit elevated PTH levels with mild-to-moderate hypercalcemia, which happens to be typically less pronounced than in malignant disease. The physical exam is usually ordinary, with palpable neck masses or vocal cord involvement uncommon. These help in distinguishing adenomas from more aggressive pathology such as parathyroid carcinoma [38, 40].

## 6. Imaging Characteristics

**Figure 2:**
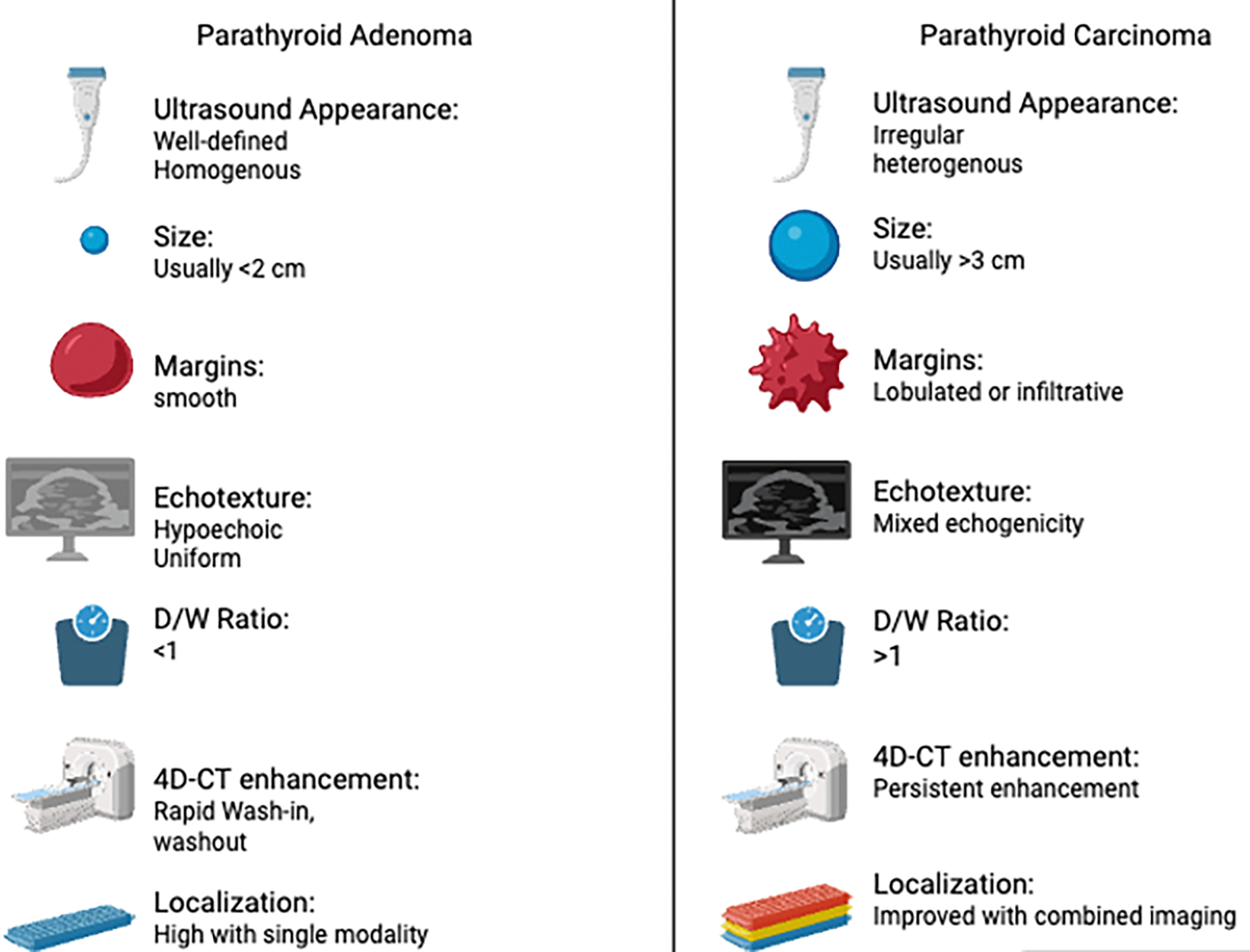
Key distinguishing features in the imaging characteristics of parathyroid adenoma and parathyroid carcinoma. D/W ratio, depth-to-width ratio; 4D-CT, four-dimensional computed tomography.

Preoperative imaging is an important step in evaluating and localizing parathyroid lesions, but distinguishing parathyroid carcinoma from adenoma can be difficult. Preoperative imaging typically includes ultrasound, sestamibi scintigraphy, and four-dimensional computed tomography (4D-CT). Ultrasound is one of the first imaging studies used to evaluate a suspected parathyroid mass. Parathyroid adenomas typically present as small, well-defined, hypoechoic lesions on ultrasound, usually with smooth borders and a more uniform, homogeneous appearance [45–47]. They are also easier to visualize on both ultrasound and sestamibi scans, showing sensitivities of around 80% for single-gland disease [48, 49]. Another key difference is the depth-to-width (D/W) ratio on ultrasound. Adenomas typically show a ratio less than 1, and most benign lesions typically fall below this threshold [45]. Furthermore, on 4D-CT, strong arterial-phase enhancement followed by rapid washout is a classic pattern for parathyroid adenomas [48] (Figure 2). In contrast, parathyroid carcinoma tends to present as a larger and more irregular mass. These lesions are often lobulated, less well-defined, and more heterogeneous compared to adenomas. Studies show that carcinomas are significantly larger than adenomas (mean around 3.5 cm vs. 1.9 cm) and are more likely to have irregular shape, non-circumscribed margins, with calcifications [47]. Localization of parathyroid carcinoma can be achieved successfully using combined imaging studies. Using a single imaging modality, such as ultrasound, yields ~80% accuracy, whereas combined studies achieve near-complete localization [48] (Figure 2).

Other features that can be used to identify parathyroid carcinoma include local invasion, cystic changes, and slower washout on 4D-CT studies. Compared to adenomas, studies have shown that carcinomas tend to have a D/W ratio greater than or equal to 1, which can reflect the invasive nature of malignant cells [45–47]. Finding evidence of invasion into nearby structures such as the thyroid gland, strap muscles, or the recurrent laryngeal nerve greatly raises suspicion for parathyroid carcinoma [47, 48]. Studies have demonstrated that lesions larger than 3 cm are more consistent with parathyroid carcinomas than adenomas, especially when combined with other suspicious features such as central calcifications or local invasion [42]. Despite the distinct imaging features discussed above, no single imaging study can distinguish parathyroid carcinomas from adenomas. Combining imaging modalities can improve localization accuracy, but it still does not reliably differentiate carcinoma from adenoma [48]. Due to overlap in presentation, imaging studies should always be interpreted with clinical findings in mind. In cases of severe hypercalcemia with severe symptoms, it may raise suspicion of carcinoma, but this can also be seen in adenomas [38]. Markedly elevated calcium levels (≥14 mg/dL) and very high PTH levels (>5× normal) should raise suspicion for malignancy, regardless of imaging findings [39]. In some cases of hyperparathyroidism, symptoms and lab findings may be severe, so even these findings can make it difficult to clearly distinguish between the two. For these reasons, obtaining a definitive diagnosis before proceeding with surgery can be difficult [26].

## 7. Intraoperative Findings

Intraoperative assessment is one of the most crucial opportunities to distinguish parathyroid carcinoma from adenoma [50]. The findings provide the surgeon with key information to determine which resection approach to use. The decision to perform en bloc resection versus a limited parathyroidectomy often depends on the surgeon’s level of suspicion at the time of surgery [50, 51]. Initial surgery provides the best chance for complete tumor removal and long-term disease control, so early recognition of the distinct characteristics of parathyroid carcinoma is critical [39–41, 26]. Parathyroid adenomas are typically soft, mobile, and well-encapsulated, with a reddish-brown appearance [39, 45]. On the contrary, parathyroid carcinomas are usually firm, adherent, and gray-white in appearance. In parathyroid carcinomas, there is also usually fibrosis and possible invasion of surrounding structures, such as the thyroid gland, strap muscles, or the recurrent laryngeal nerve [39, 26, 45]. Just as other features can overlap, intraoperative findings can also overlap and not present in a straightforward manner [50]. Some large adenomas can appear suspicious and mimic carcinoma. Studies have shown that several patients had intraoperative findings concerning carcinoma, but only a portion were confirmed as malignant on final pathology [26, 50].

## 8. Risk of Capsular Rupture

Parathyroid carcinomas have a much higher risk of capsular rupture due to their anatomical features and the biological nature of malignant cells [51]. Carcinomas are more likely to invade and adhere to nearby structures, making dissection more complex and requiring careful handling. Capsular rupture can lead to tumor dissemination and increase the risk of local recurrence and parathyromatosis [51, 52]. For this reason, if carcinoma is suspected intraoperatively, a standard parathyroidectomy should be avoided [52]. En bloc resection has been shown to significantly reduce the risk of recurrence compared with limited approaches [40].

## 9. Importance of Intraoperative Suspicion

So far, no single pre- or intraoperative finding reliably distinguishes the two due to overlapping presentations. The best approach is to use preoperative factors such as very high PTH levels (>5× normal), calcium ≥14 mg/dL, large tumor size, and elevated alkaline phosphatase combined with imaging studies and intraoperative findings to gauge the level of suspicion [51]. This should aid the surgeon in determining whether to proceed with en bloc resection, given the likelihood of carcinoma. En bloc resection allows removal of the tumor, the ipsilateral thyroid lobe, as well as any involved surrounding tissue [39–41, 26].

## 10. Surgical Techniques for Initial Resection

Parathyroid carcinoma is a rare and aggressive endocrine malignancy, accounting for less than one percent of primary hyperparathyroidism cases [22]. As a result, diagnosis should be considered in patients presenting with primary hyperparathyroidism, elevated parathyroid hormone (PTH) levels, and severe hypercalcemia [22]. Given the aggressive nature of this malignancy, complete surgical excision remains the primary treatment, as incomplete tumor removal substantially increases the risk of recurrence. For suspected or confirmed parathyroid carcinoma, *en bloc* resection is the preferred surgical technique because it maximizes the likelihood of total tumor removal and reduces the risk of local recurrence [22]. Specifically, *en bloc* resection involves excision of the affected parathyroid gland, an ipsilateral thyroid lobectomy, and removal of adjacent tissues if invasion is present. Given that the parathyroid glands are anatomically close to the thyroid, an ipsilateral thyroid lobectomy is often performed to manage possible tumor invasion. Ultimately, the main goal is to achieve negative surgical margins and prevent capsular rupture or tumor seeding, as achieving negative margins is the most significant prognostic factor in parathyroid carcinoma [11]. En bloc resection results in margin-free excision in approximately 82% of cases, compared to only 17% with simple local excision [11]. Patients with negative margins experience excellent outcomes, with 100% recurrence-free survival at both 5 and 10 years, whereas positive margins are associated with local recurrence in approximately 50% of cases [11]. In cases of recurrent disease, extended en bloc resection further improves outcomes, with a 5-year overall survival rate of approximately 59.6%, compared to 16.7% following less aggressive surgical interventions [11].

In addition to the extent of resection, the presence of local invasion increases the complexity of surgical management. Parathyroid carcinoma frequently invades adjacent cervical structures, such as the strap muscles, thyroid capsule, and surrounding connective tissue [55]. When invasion is suspected or identified intraoperatively, resection of the involved soft tissues is recommended as part of the en bloc approach [15]. Preservation of tumor capsule integrity is essential, as rupture can lead to tumor spillage and seeding, significantly increasing the risk of recurrence [15]. The American Association of Endocrine Surgeons recommends complete resection while avoiding capsular disruption to improve the likelihood of cure [22]. Capsular disruption may lead to parathyromatosis, where tumor cells seed throughout the operative field, making definitive cure extremely challenging [52]. Therefore, fine-needle aspiration is contraindicated due to the risk of tumor cell dissemination [52]. If capsular rupture occurs, prognosis worsens considerably, with recurrence often developing within 6 months to 3 years after surgery, frequently indicating disease that is difficult or impossible to cure [52].

This aggressive surgical approach differs considerably from the management of benign primary hyperparathyroidism, where a standard parathyroidectomy is typically sufficient. In fact, approximately 85% of primary hyperparathyroidism cases stem from a single parathyroid adenoma and removing that gland alone is generally curative [22]. For these patients, minimally invasive parathyroidectomy (MIP) has become the preferred approach, as it limits dissection, reduces postoperative pain, and allows for a faster recovery [22]. Most MIP procedures rely on preoperative localization imaging to guide the operation, making them far less extensive than what is required for malignant disease [22]. The difference in approach between benign and malignant parathyroid disease reflects a broader principle: when carcinoma is suspected, oncologic control takes priority over minimizing surgical exposure. This distinction also applies to emerging technologies in endocrine surgery. While robotic and AI-assisted techniques have gained traction in benign parathyroid disease, where lesions are well localized and a minimally invasive approach is feasible, their role in parathyroid carcinoma is much more limited [69]. The wide surgical exposure, en bloc resection, and careful tumor handling required in malignant disease make these technologies difficult to apply in practice [22]. As a result, open surgery guided by intraoperative clinical judgment remains the standard of care for parathyroid carcinoma.

## 11. Indications and Contraindications of Resecting Parathyroid Carcinoma

The indications for *en bloc* resection in parathyroid carcinoma span the preoperative, intraoperative, and pathological domains, and recognizing these factors is essential for guiding surgical decision-making. According to the AAES guidelines, when intraoperative suspicion for parathyroid carcinoma arises, complete resection should be performed while carefully avoiding capsular disruption, which may necessitate en bloc resection of adherent tissues [22]. Preoperatively, indicators of malignancy include serum calcium greater than 14 mg/dL, a palpable neck mass, intact parathyroid hormone (iPTH) levels exceeding 8 times the upper limit of normal, or suspicious and irregular margins on ultrasonography [6]. The clinical impact of preoperative suspicion is significant. When present, 10 of 11 patients underwent en bloc resection; in its absence, only pericapsular resection was performed in 6 of 9 cases [6]. Intraoperatively, findings of local invasion, including adherence to or invasion of the thyroid, trachea, esophagus, recurrent laryngeal nerve, or strap muscles, further support the need for en bloc resection. Notably, parathyroid carcinoma adheres to adjacent structures in 77.8% of cases, compared to just 20.6% for atypical adenomas [25]. Gross tumor characteristics such as a gray-white, stone-hard appearance, absence of a distinct capsule (47.1% of carcinomas versus 12.9% of atypical adenomas, P = 0.03), and tumor size of at least 3 cm also serve as important intraoperative cues [58,57]. In the setting of recurrence, extended *en bloc* reoperation (EEBR), which removes all tumor-bearing scar tissue, achieves a 5-year overall survival (OS) rate of 59.6% and local control in 84% of patients, compared to only 16.7% 5-year OS with less radical procedures [12].

In contrast, limited resection, such as parathyroidectomy, may be appropriate in select cases. When the tumor is completely encapsulated with no evidence of invasion into neighboring structures, a focused parathyroidectomy is preferred. This is most seen in benign adenomas presenting with mild hypercalcemia and no palpable mass, which intraoperatively appear soft, well-circumscribed, and easily separated from surrounding tissues [6, 55]. Of note, if carcinoma is not initially suspected, reoperation is considerably more challenging and less effective. Data from the California Cancer Registry show 5- and 10-year survival rates of 86.9% and 72%, respectively, with no significant difference in survival between parathyroidectomy alone, en bloc resection, or delayed thyroid resection; only age and distant metastases independently predicted survival [59]. Patients with small, well-encapsulated tumors lacking vascular invasion may similarly be candidates for limited resection, consistent with low-risk Schulte staging [59]. That said, limited resection carries a 3.5-fold higher risk of positive margins and a 6.4-fold higher risk of locoregional recurrence compared to *en bloc* resection [11]. For this reason, limited resection is only justified when the diagnosis was unsuspected, and the tumor was completely excised with an intact capsule and negative margins [11].

### 11.1. Absolute Contraindications

Although there are no absolute contraindications specific to parathyroid carcinoma, the extent of surgical intervention should be carefully considered. En bloc resection is warranted only when it can realistically achieve complete tumor removal and provide a meaningful survival benefit. Widespread distant metastases represent an absolute contraindication to surgery [68]. The most common metastatic sites are the lungs, liver, and bones, and systemic disease in these locations does not improve with removal of the primary tumor [68]. Disease progression and hypercalcemia are often driven by metastatic burden. Unresectable local disease, such as tumor encasement of the carotid artery or major mediastinal vessels, also precludes the possibility of achieving negative margins and is associated with high recurrence and morbidity [68]. Additionally, in medically unfit patients, such as those with severe cardiopulmonary disease or high anesthesia risk, the risks of surgery outweigh the potential benefits.

### 11.2. Relative Contraindications

Beyond these absolute contraindications, several relative contraindications also warrant careful consideration. Each case must be evaluated individually, weighing the risks of surgery against the potential for meaningful benefit. In the setting of limited oligometastatic disease, surgery should only be pursued if both the primary tumor and metastases are resectable or if the patient is symptomatic. In some cases, cytoreductive procedures may still be useful for controlling hypercalcemia [61]. Similarly, recurrent disease after prior surgery poses a relative contraindication, as a scarred surgical field significantly increases the risk of complications [60]. While surgery may still be considered for localized recurrence or in symptomatic patients, the likelihood of cure is lower than with the initial operation [60, 61]. Further complicating reoperation is the risk of injury to critical structures, such as the recurrent laryngeal nerve, the strap muscles, or the thyroid lobe [62]. Unilateral nerve injury can cause hoarseness and dysphonia, whereas bilateral involvement can lead to serious airway compromise, risks that may outweigh the potential benefit of surgery [62, 63]. When negative margins and complete tumor removal are no longer achievable, surgery becomes noncurative, and the focus should shift towards palliative care.

## 12. Role of Intraoperative Findings

With these contraindications in mind, preoperative imaging plays an important role in surgical planning, particularly for assessing the primary lesion, evaluating local invasion, and identifying recurrent or metastatic disease [70]. However, imaging alone cannot reliably distinguish parathyroid carcinoma from benign primary hyperparathyroidism in all cases, and operative planning should ultimately be guided by biochemical severity, physical findings, and intraoperative assessment [70].

Given that preoperative diagnosis is achieved in only 25% of cases, intraoperative assessment becomes critical for surgical decision-making [64]. Macroscopic features raising concern for malignancy include a firm gray-white mass, adhesion to the thyroid or strap muscles, loss of a distinct capsule, and a fibrotic or desmoplastic reaction [64]. Among intraoperative events, tumor rupture is the most serious, as both rupture and capsular violation are strongly associated with recurrence and disease-specific mortality and must be avoided at all costs [65]. Intraoperative PTH monitoring can further guide the extent of resection. A drop of more than 50% into the normal range is a strong indicator of surgical success, with roughly 93% of patients remaining normocalcemic at 6 months [4]. If the PTH falls by more than 50% but stays above normal, success is less reliable, with only about 50% of patients achieving normocalcemia [4]. Recurrent laryngeal nerve neuromonitoring is also worth employing, as it significantly reduces the rate of voice changes related to the external branch of the superior laryngeal nerve compared to cases without monitoring (12.5% vs. 57.14%) [65]. Finally, frozen section analysis should not be relied upon to determine the extent of resection, as it correctly identifies carcinoma in only about 15% of cases [3].

## 13. Role of Lymph Node Dissection

Lymph node dissection plays a limited but important role in the surgical management of parathyroid carcinoma. Because lymphatic spread occurs less frequently than local tissue invasion, routine dissection is not advised; rather, lymph node removal is reserved for cases with clinical or intraoperative evidence of nodal involvement [55]. Consistent with this approach, the American Association of Endocrine Surgeons (AAES) guidelines state: “Prophylactic central or lateral neck dissection should not be performed for parathyroid carcinoma” [22]. While lymph node metastases are uncommon overall, occurring in 6.5% of 972 published cases, the rate increased substantially to 32.1% among 196 patients when lymph nodes were formally assessed [55]. Similarly, a SEER database analysis reported 10.5% positive nodes in 114 patients who underwent lymph node examination. These findings indicate that formal assessment likely underestimates true nodal involvement. Notably, risk factors for nodal metastasis include tumor size of at least 3 cm, high-risk Schulte staging, and CDC73 abnormalities, all of which are statistically significant predictors [56, 57]. Given these risk factors, lymph node dissection may be considered in patients with high-risk features [56, 57].

With respect to anatomic distribution, the central compartment of the neck is typically the initial site of spread when lymph node metastasis is suspected. Lateral neck dissection, by contrast, is less frequently required and is considered only when imaging or intraoperative findings indicate metastasis to the lateral cervical lymph nodes. Supporting this pattern, retrospective analyses demonstrate that nodal metastasis is uncommon and primarily involves the central neck compartment [55]. Furthermore, recurrence is more often in the surrounding soft tissues than in lymph nodes [55]. Taken together, these results indicate that *en bloc* resection with removal of potentially involved soft tissues and central compartment lymph nodes may improve outcomes, whereas routine prophylactic lateral neck dissection remains unnecessary [55]. Schulte et al. similarly concluded that systematic central lymph node resection as part of *en bloc* clearance may improve outcomes, but emphasized there is no role for prophylactic lateral neck dissection [55]. Importantly, SEER analyses demonstrate that positive lymph node status is not an independent prognostic factor for disease-specific survival on multivariate analysis [56].

A key aspect of parathyroid carcinoma management is the high rate of locoregional recurrence and the necessity for lifelong surveillance, both of which further explain the limited role of routine lymph node dissection [66]. Recurrence occurs in 30–75% of patients, most often within the operative bed or adjacent soft tissues rather than in lymph nodes or distant sites [66]. Incomplete initial resection often drives this recurrence, stressing the importance of achieving negative margins during the first operation for long-term outcomes. Given this risk, biochemical monitoring is central to follow-up, as rising serum calcium and parathyroid hormone (PTH) levels often precede clinical or radiographic evidence of recurrence and serve as sensitive early indicators of disease relapse [71]. As a result, patients require lifelong surveillance with periodic calcium and PTH measurements, while imaging is reserved for cases of biochemical recurrence to localize disease. When recurrence is identified, repeat surgical resection may be considered, particularly for symptomatic hypercalcemia, although cure rates decrease and morbidity increases with each subsequent operation [71]. Collectively, this recurrence pattern further supports the view that parathyroid carcinoma is a locally aggressive endocrine malignancy, where local control and biochemical management are prioritized over prophylactic lymph node clearance [71].

Due to the significant risk of delayed locoregional recurrence, patients with parathyroid carcinoma require lifelong biochemical surveillance following surgery [68]. Specifically, follow-up focuses on serial serum calcium and PTH measurements, as biochemical recurrence frequently precedes radiographic detection [68]. Consequently, imaging is generally reserved for patients with rising calcium or PTH levels or when there is clinical suspicion of recurrence. Ultimately, this long-term follow-up approach acknowledges that recurrence may occur years after initial resection and that effective disease control relies on early identification of recurrent hyperparathyroidism. During resection of parathyroid carcinoma, monitoring intraoperative parathyroid hormone (IOPTH) levels is common practice. Since parathyroid hormone levels have a short half-life, around three to five minutes, IOPTH is used to determine if hypersecreting tissues have been excised [72, 73]. In parathyroid adenoma resections, a decrease of >50% of baseline PTH levels in 10–15 mins is usually an adequate decrement, and hence the surgeons can avoid further exploration [74]. However, in parathyroid carcinoma, this criterion is insufficient because malignant tumors can produce large amounts of PTH, and residual microscopic disease/metastatic tissue continues to produce PTH (even in the absence of the primary tumor) [6, 68]. IOPTH can determine that the dominant tumor was excised, but it cannot determine the absence of metastasis and thus cannot deviate from surgical oncology [75, 76]. In recurrent/persistent parathyroid carcinoma, the interpretation of intraoperative PTH levels is expected to be most challenging due to multiple gland involvement, metastases, or altered physiology due to prior surgery [77, 78]. Most of the latest reviews suggest that IOPTH monitoring should be viewed as an additional aid that may assist the surgeon’s intraoperative decision-making, but does not establish definitive surgical success in carcinoma, of which there still exists complete margin-negative resection as the principal factor for determining long-term control [11, 79].

Recurrent laryngeal nerve (RLN) monitoring is often used during surgery on the parathyroid glands as an adjunct intraoperatively, and parathyroid carcinoma carries a higher risk of RLN injury because patients may have had a larger surgical resection because of local tumor invasion. Parathyroid carcinoma may have invaded and/or adhered to adjacent tissues and organs, including the thyroid gland, strap muscles, and the RLN. This makes nerve identification and preservation of the RLN an important part of the operative management [11, 80, 81]. Intraoperative nerve monitoring (IONM) provides surgeons with real-time feedback regarding the functional integrity of the RLN, allowing surgeons to identify the RLN during surgeries in distorted anatomy and during re-operative procedures when the anatomy is grossly distorted due to prior surgical scarring [68, 82]. The importance of IONM intervention in parathyroid carcinoma cannot be overstated, as re-operative procedures following parathyroidectomy are common and are associated with a higher risk of RLN injury due to the high rates of recurrence following initial surgery [61, 83].

The clinical limitations of IONM include not being able to replace anatomic understanding or careful dissection techniques. IONM serves primarily as an adjunct to visual identification but does not adequately prevent nerve injuries when a tumor surrounds the RLN, and hence, the RLN must be removed in cases of complete oncologically acceptable resection [84, 79]. In cases where the RLN is encompassed by the tumor, preservation may be oncologically inappropriate; thus, an *en bloc* resection involving the RLN may be necessary to achieve margin-negative excision, which remains the most significant indicator of long-term disease control [11, 72]. There can also be false positives or false negatives with nerve monitoring, making it a less reliable indicator of nerve function because of prior surgical manipulation or scarred tissue in the area being monitored [82]. Overall, there are numerous studies supporting the use of intraoperative neuromonitoring as an appropriate adjunct to complex, revisional parathyroid carcinoma surgery, but make it clear that oncologic surgical principles should override nerve preservation when there is a conflict between nerve preservation and the complete removal of the tumor [68, 79].

Parathyroid biopsy from frozen section analysis is used in operations done on the parathyroid glands with the primary goal of confirming that the tissue being removed is indeed from a parathyroid gland. Use of this technology in diagnosing parathyroid cancer is limited and should be used cautiously. When there is uncertainty regarding the diagnosis of tissue, frozen sections can be helpful in differentiating between a parathyroid gland, a thyroid gland, a lymph node, or other neck structures, but will not have an impact on surgical decision-making in most cases [28, 84]. The frozen section is not effective in distinguishing between benign and malignant parathyroid adenoma and carcinoma. The definitive histopathologic features of carcinoma, such as capsular invasion, vascular invasion, and perineural invasion, will not be found with sufficient definition upon examination of frozen section and require careful examination after permanent sectioning [79, 84].

In patients with parathyroid carcinoma, the decision to do an *en bloc* resection is often dependent upon intraoperative evaluation, such as finding a firm, adherent mass that shows local invasion, versus using only results from frozen sections for guidance [68, 75]. It has been shown that early recognition of potentially suspicious features and timely *en bloc* resection can have a positive effect on patient outcomes, but if only a limited excision is made and the carcinoma is discovered at a later time, this will greatly increase the chances of the disease recurring and needing to undergo additional reoperation [11, 85]. The frozen section should not be utilized independently of clinical judgement or established principles of oncologic surgery [79, 84].

The function of frozen section pathology in the setting of parathyroid surgery is mainly to provide a tissue type identification on the path report for parathyroid tissue. Presently, there are very few applications for the use of a frozen section to assist in providing a diagnosis or encouraging a diagnosis of parathyroid carcinoma. In some cases, it may not be possible to determine the tissue type definitively before a frozen section. In these instances, a frozen section can differentiate between parathyroid tissue and thyroid tissue, lymph nodes, and other cervical structures, which can assist with intraoperative decision-making [28, 84]. Although a frozen section may assist with tissue identification, it does not have sufficient sensitivity or specificity to provide definitive diagnosis of a benign or malignant parathyroid gland, because the consistently assessed key histopathologic characteristics of parathyroid gland carcinoma, including vascular invasion, capsular invasion, and perineural invasion will generally not be able to be assessed in a frozen section, and the diagnosis of parathyroid carcinoma will generally require completion of the permanent pathological or histopathologic evaluation [79, 84].

Three intraoperative adjuncts can help surgeons improve outcomes in parathyroid carcinoma surgeries. These adjuncts include IOPTH monitoring, RLN monitoring, and frozen section pathology. While each of these adjuncts provides specific benefits, they also have significant limitations that may restrict their role in guiding definitive management. IOPTH monitoring can give real-time biochemical feedback during surgery, which can help the surgeon confirm that a major source of PTH secretion has been respected from the patient’s body. This benefit is especially useful in complex or re-operative cases [72, 73], but the reliability of IOPTH monitoring is diminished for patients with parathyroid carcinoma because residual microscopic disease or metastatic deposits may still secrete PTH, even if the intraoperative PTH level has dropped significantly [80,683] Similar to IOPTH monitoring, the use of RLN monitoring during surgery improves nerve identification and provides functional feedback during dissection; thus, this adjunct is very helpful during surgery to resect parathyroid carcinoma, particularly when the tumor is very invasive, making it difficult for the surgeon to identify the anatomic landmarks [82,68]. However, RLN monitoring does not prevent nerve injury from occurring, nor does it alter oncologic priorities; when the tumor is located on or involving an RLN, the nerve will have to be sacrificed to complete the tumor resection [11, 79, 84].

Frozen section pathology can confirm whether excised tissue is parathyroid in origin and helps distinguish parathyroid from normal structures immediately adjacent to the excised tissue [28, 84]. However, the diagnosis of parathyroid carcinoma cannot be reliably determined from frozen section pathology, specifically, because of key characteristics of malignancy. An example of this would be capsular and/or vascular invasion, which are not typically identifiable on frozen section specimens; the diagnosis of parathyroid carcinoma based solely on frozen section pathology is inadequate to direct oncologic decision-making [79, 84].

## 14. Special Surgical Scenarios

Parathyroid carcinoma has a high degree of local invasion, which greatly impacts both surgical treatment and prognosis. Tumors are typically much firmer and adhere to surrounding tissues than benign parathyroid adenomas, with palpable neck masses often presented [85]. Examples of this can be seen with thyroid, strap muscles, recurrent laryngeal nerve, trachea, esophagus [75, 79, 80]. The inability to mobilize the tumor or its adherence to the surrounding tissues is suggestive of an invasive tumor at surgery [68, 84]. The pathological diagnosis of invasion is made histologically by evidence of capsular invasion, vascular invasion, and perineural invasion [79, 84].

Localized invasion of cancer calls for an aggressive surgical approach, usually consisting of total *en-block* tumor resection and removal of all relevant adjacent structures to obtain margin negativity [11, 75]. In addition, this often requires ipsilateral lobectomy, removal of muscular invasion, and possibly sacrificing the right RLN, whose anatomy is involved due to tumor infiltration [11, 77]. Inadequate identification or treatment of local invasion at the first surgical procedure has been consistently associated with a dramatically higher risk for the patient to develop persistent or recurrent disease and ultimately results in a more complicated reoperation with added risk for morbidity [61, 76, 78]. Containing only margin-negative resections at the initial surgical procedure is consistently identified as the only significant prognostic factor for long-term tumor control and overall survival; limited re-excision without margin-negative resections can provide the opportunity for seeding and early recurrence of the tumor [61, 77, 79].

The presence of vascular invasion is what defines parathyroid carcinoma on the histopathologic level, such as the microscopic characteristics of the tumor, and is one of the most important criteria for distinguishing a malignant form of parathyroid disease from a benign, an example of which being an adenoma, a form of parathyroid disease. Malignancies like Parathyroid carcinoma tumor cells that invade into or through the walls or lumens of surrounding blood vessels, as well as through the capsule of the tumor and into the perineural space, are a very reliable indicator of malignancy [79, 86]. Importantly, vascular invasion is identified when examining the tumor specimen using permanent histopathologic techniques rather than during the operation to remove the tumor; therefore, vascular invasion cannot be visually identified during gross inspection of the specimen or on frozen section analysis [84]. Vascular invasion also provides insight into the biological aggressiveness of the tumor and its capacity for dissemination from the primary tumor; therefore, vascular invasion contributes to the occurrence of local recurrence and the development of distant metastases.

From a clinical point of view, when vascular invasion is present, there is a greater risk that a patient will have recurrence of their disease, as well as poorer long-term results because vascular invasion is a means by which the cancer spreads through the bloodstream to distant sites like the lungs, bone, and liver [12, 23]. Although the fact that vascular invasion is present does not change the intraoperative decision-making process in real-time, it does make clear how critical it is that the first resection of the tumour be performed as an *en bloc* resection with negative margins to limit the possibility of residual disease and spread of the cancer to other parts of the body [11, 75]. In addition, finding vascular invasion on final pathology often results in closer postoperative monitoring, as recurrence of the cancer is now more likely to occur years after the original surgery [12, 23]. Therefore, while vascular invasion is mainly a postoperative finding, it has major clinical implications for the prognosis of a patient with parathyroid carcinoma, as well as the follow-up and overall management of the patient's disease [72, 79].

## 15. Recurrent and Persistent Parathyroid Carcinoma

Persistent and recurrent disease in parathyroid carcinoma is classified based on the timeline of hypercalcemia following initial operative management, which is an important aspect of perioperative care management and subsequent reintervention strategies. Persistent disease is defined as the failure to establish normocalcemia after surgery, or, in the case of return to hypercalcemia within the first six months post-operatively, suggesting residual tumor tissue likely remains from the initial operation [29, 61]. Recurrent disease is defined as the return of hypercalcemia after having been in documented normocalcemia for a minimum of six months, suggesting either tumor regrowth or development of new metastatic deposits [12, 61]. These definitions are utilized routinely in both clinical practice and the literature to differentiate between incomplete initial resection and true recurrence of disease.

The distinction between recurrent and persistent diseases carries significant implications for the management of parathyroid carcinoma patients. Persistent disease represents inadequate initial surgical treatment, such as incomplete excision; previously established locally invasive carcinoma, while recurrence of the disease is a consequence of the natural course of an aggressive malignancy despite appropriate initial treatment [11, 85]. Persistent disease often triggers early reoperation if an identifiable localized source is detected; however, recurrent disease necessitates a more complex, staged treatment modality including numerous repeat surgical procedures over time to manage hypercalcemia and tumor burden [61, 87]. Because of the high rates of recurrence for parathyroid carcinoma and potential for late relapse, definitions for both types of disease also help guide long-term surveillance protocols that include continued biochemical follow-up many years following initial treatment [12, 23].

Patients with parathyroid cancer have a high level of recurrence, estimated to be around 40–60% after the first surgical intervention [12, 23]. Most recurrences happen within the first two to four years of surgery; however, there are also many reports of patients developing a recurrence greater than ten years after surgical intervention, indicative of the slow but continued growth of the cancerous body [12, 21, 23]. Early recurrences are more likely to occur in patients with more aggressive disease; therefore, late recurrences may occur as a function of the slow-growing residual microscopic disease [23]. Due to this variation, biochemical surveillance should last indefinitely to ensure biochemical testing for calcium and parathyroid hormone levels.

Most of the time, the way people develop hyperparathyroid disease is because the original tumors were not completely removed or they ruptured [61, 78]. Patients with advanced or returning hyperparathyroid disease can have the original tumors extending beyond their original locations into nearby lymph nodes throughout the neck and chest [14, 16]. Distant metastases, such as those that develop in the lungs, bones, or liver, are less common [12, 79]. When these patients develop local recurrence, it is usually because they have been treated again through surgical procedures designed primarily to control hypercalcemia and reduce the burden of disease rather than to achieve a cure for the patient. After all, recurrent disease tends to progress and become chronic over time [61, 74].

Parathyroid carcinoma reoperation is typically performed when the patient has ongoing/recurrent illness with symptomatic/biochemically significant hypercalcemia. It can also occur when there are severe complications associated with hypercalcemia, such as but not limited to: nephrolithiasis, renal dysfunction, neurocognitive symptoms, and cardiac abnormalities. To combat this, surgical intervention would still be warranted to attempt to treat hypercalcemia by reoperation because of the potentially serious consequences of hypercalcemia and the need to provide relief from said symptoms [68, 7, 61]. In addition, when there is a clearly defined disease site on imaging, it may be a candidate for re-operative intervention. An example of this is a recurrent disease found in the neck, mediastinum, or having isolated metastasis. If the tumor can be surgically removed, it would also be a candidate for reoperative intervention, as it may provide a means for reducing the tumor burden as well as providing better control of the biochemistry of hypercalcemia [9, 78, 85].

Additional criteria indicating progression can be demonstrated with imaging studies, even with initially mild symptoms. This is shown when there is an increase in calcium levels or evidence of rising levels of PTH over time. Therefore, advanced tumor progression may be indicated [12, 23]. Reoperation is done primarily for palliative purposes to achieve control over the hypercalcemia and to prevent future complications, rather than for a potential cure, especially in the case of patients presenting with either metastatic or multifocal disease [61, 86] Careful consideration must be given to patient selection for reoperation due to the increased risk of complications associated with the presence of scar tissue, such as anatomical distortion, and the higher incidence of complications, such as recurrent laryngeal nerve injury. [61, 78]. Accordingly, reoperation is reserved only for patients in which the benefit derived from biochemical control and improving symptoms outweighs the associated risks of the procedure, with the disease localization accurately determined to permit targeted intervention [68, 78].

When it comes to the surgical treatment for recurrent parathyroid carcinoma, the goal is to surgically remove all possible signs of the disease to help manage hypercalcemia and lessen the burden caused by the tumors. In recurrent cases, the disease is typically located in the cervical, thus if imaging of the affected area demonstrates a present tumor, the best practice would be to try to remove the tumor and any structures that have localized disease during surgery by performing “en bloc” [78, 87]. This consists of removing everything together as one piece, essentially a total tumour resection, which includes surrounding straps of muscles and thyroid glands and, when appropriate, may require sacrificing the recurrent laryngeal nerve, due to the tumor, to obtain negative surgical margins [11, 77]. In cases where there is a recurrence of the tumor in other areas of the body, a larger surgical approach may be required, such as using the thorax or sternum, to reach the diseased area to surgically remove the disease [84, 88]. Unfortunately, since patients will have multifocal disease, it is unlikely that they will be able to be cured. Therefore, if there are recurrences after multiple surgeries have been completed, follow-up procedures will follow. There is a pattern where if there are inaccessible tumor sites or missed tumors post initial surgery, reoperation, often via a sternotomy, is required for successful treatment [89].

Before surgery that requires operations on a different site, the physician must obtain accurate preoperative localization of the parathyroid tumors to perform the surgery. Several different types of imaging have been used to locate the sites of the functional (adenomatous) parathyroid tissue, including sestamibi scanning, CT scan, MRI scan, and selective venous sampling for parathyroid hormone (PTH) levels [86]. There is an emphasis on the removal of any dominant or symptomatic lesions that cause hypercalcemia while leaving small/accessible metastases if present [61, 86]. There is significant concern about potential complications after reoperation of the cervical region due to the potential for damage to the surrounding structures, such as nerves. When undergoing a procedure, it is imperative to carefully plan so that morbidity can be minimized while also maximizing disease control [61, 86]. Overall, the data suggest a personalized and sequential surgical approach for patients with parathyroid carcinoma experiencing a recurrence, that repeated resections may provide chronic disease management and long-term biochemical control of the parathyroid disease process [86, 87].

The purpose of reoperation in the treatment of parathyroid cancer is to manage high calcium levels and to remove as much tumor as possible, but not to cure the cancer. Although cure rates are still lower than after initial surgery, patients can still have their calcium levels lowered, and some will even become asymptomatic from the same surgery [61, 86]. Complete remission after reoperation is rare in patients with multifocal or metastatic disease. However, patients who have undergone selective removal of dominant lesions often have a better quality of life due to their prolonged periods of disease control. [61, 78]. Outcomes of long-term surgery vary, but the nature of parathyroid carcinoma is persistent, and this can be seen with the poor prognosis following reoperation. Five-year survival rates are relatively high (>80%); however, recurrence occurs frequently, necessitating additional surgical interventions over time [86, 87]. Patients frequently undergo repeated surgeries during their disease, with each surgery providing incremental control of hypercalcemia rather than a cure [61, 86]. Re-operative surgery is associated with a higher complication rate because of scar tissue formation and the distorted anatomy from previous procedures [61, 78]. Re-operative surgery is an important part of managing the long-term course of parathyroid carcinoma, both in terms of palliation of symptoms and controlling the disease, given that the disease recurs at high rates and progresses slowly.

## 16. Adjuvant and Non-Surgical Therapies

### 16.1. Radiation Therapy: Evidence and Ongoing Controversy

Parathyroid carcinoma has historically been considered relatively radioresistant, and the role of adjuvant external beam radiation therapy (EBRT) has remained controversial. Earlier retrospective studies concluded that routine adjuvant radiation following surgical resection was not standard practice, because disease-free survival and overall survival showed definitive improvement. These conclusions were largely based on small institutional series and heterogeneous retrospective datasets [75, 77, 80]. Despite this, several reports suggested that EBRT may reduce local recurrence rates, particularly in high-risk settings. The M.D. Anderson experience demonstrated lower rates of localized recurrence among patients who received adjuvant radiation following initial surgery, independent of tumor stage or extent of resection, even though patient numbers were small and selection bias was unavoidable [90]. Similar findings were supported by subsequent institutional series and interdisciplinary reviews, leading some authors to support the selective use of EBRT rather than completely avoiding it [7, 68, 75, 90].

More recently, emerging population-level data have challenged the historical assumption that adjuvant radiation provides no survival benefit. A propensity score-matched analysis of the Surveillance, Epidemiology, and End Results (SEER) database demonstrated significantly improved disease-specific survival among patients receiving adjuvant radiation therapy. In that study, 10-year disease-specific survival was 90.3% in the radiation cohort compared with 72.2% in patients managed without radiation (p=0.02), with this difference persisting at 20 years (90% vs. 68%, p=0.03). On multivariable analysis, omission of adjuvant radiation was an independent predictor of worse disease-specific survival (hazard ratio 3.2, 95% CI 1.3–10.1) [2].

In contrast, a prior National Cancer Database analysis did not demonstrate an overall survival benefit of adjuvant radiation therapy, underscoring the persistent difference in retrospective database studies and highlighting the limitations inherent to such analyses [8]. Accordingly, while routine use of adjuvant radiation cannot yet be universally recommended, current evidence supports considering the use of EBRT in select high-risk patients, including those with positive or close surgical margins, gross extrathyroidal or soft-tissue invasion, unresectable localized disease, or multiple local recurrences. Experts recommend individualized decision-making within a multidisciplinary setting [7, 75, 80, 91].

### 16.2. Medical Management of Hypercalcemia

For patients with unresectable, recurrent, or metastatic parathyroid carcinoma, morbidity and mortality are driven primarily by hypercalcemia rather than tumor burden. Medical therapy, therefore, plays a central role in symptom control to provide relief. Acute hypercalcemic crises require inpatient management with aggressive intravenous hydration, loop diuretics, calcitonin, and intravenous bisphosphonates [77, 75, 68]. However, most patients ultimately require long-term outpatient management of chronic hypercalcemia [80, 7, 91]. Several historical pharmacologic approaches, including estrogen-based therapies such as hexestrol, have been described in older hyperparathyroidism literature, primarily in benign disease contexts, but lack disease-specific evidence in parathyroid carcinoma and have been avoided in favor of more effective contemporary agents [80, 7].

### 16.3. Calcimimetics: Beyond Basic Cinacalcet Use

Cinacalcet, a calcium-sensing receptor agonist, represents the foundation of chronic medical management for hypercalcemia in parathyroid carcinoma. By increasing the sensitivity of the calcium-sensing receptor on parathyroid cells, cinacalcet suppresses parathyroid hormone (PTH) secretion and lowers serum calcium levels. While it does not change the disease state, cinacalcet has demonstrated durable biochemical control and symptomatic improvement in many patients with persistent or metastatic disease [77, 80, 91].

Drug dosing regimens typically involve gradual titration of cinacalcet to achieve calcium control while minimizing gastrointestinal side effects, including nausea, vomiting, abdominal pain, and diarrhea. Long-term therapy is frequently required, and dose increases over time are common due to progressive disease burden. Importantly, cinacalcet does not reduce tumor size and should be viewed explicitly as palliative rather than curative therapy [7, 75]. Recent literature has expanded on combination approaches for refractory hypercalcemia. Denosumab, a monoclonal antibody targeting receptor activator of nuclear factor kappa-B ligand (RANKL), has been used in combination with cinacalcet in patients who fail to achieve adequate calcium control with calcimimetics alone. Case reports and small series suggest additive calcium-lowering effects, particularly in patients with extensive skeletal involvement or bisphosphonate-refractory disease, although sustained long-term control remains limited [22, 91].

### 16.4. Bone Resorption Inhibitors: Palliative Role

Bisphosphonates and denosumab play an adjunctive role in the management of hypercalcemia by inhibiting osteoclast-mediated bone resorption. These agents may provide temporary reductions in serum calcium levels and symptomatic relief, particularly in patients with skeletal metastases or high bone turnover [3, 5, 9]. However, their effects are temporary, and diminishing efficacy with repeated administration is well documented. As such, bone resorption inhibitors should be regarded as palliative therapies rather than definitive treatments and are most appropriately used as part of a multimodal strategy for symptom control in advanced disease [7, 91, 22].

### 16.5. Chemotherapy and Targeted Systemic Therapies

Conventional cytotoxic chemotherapy has demonstrated minimal and inconsistent success in parathyroid carcinoma. Regimens incorporating dacarbazine, cyclophosphamide, fluorouracil, doxorubicin, lomustine, and methotrexate have been described in isolated case reports, with responses that are typically partial and short-lived [7, 77, 80]. Consequently, chemotherapy is not routinely recommended and should be reserved for highly selected cases with appropriate patient counseling. In contrast, emerging targeted therapies have gained increasing attention. Recent reports describe the use of tyrosine kinase inhibitors (TKIs), including lenvatinib, anlotinib, and surufatinib, in patients with metastatic or refractory parathyroid carcinoma, primarily supported by isolated case reports and small series that have demonstrated radiographic or biochemical responses in select patients [91, 22]. Immune checkpoint inhibitors have also been explored in isolated cases, particularly in patients with advanced disease who show molecular features suggesting they can trigger an immune response. However, evidence remains limited to informal reports [22].

### 16.6. Criteria for Selecting Adjuvant Therapy

Given the absence of high-level prospective evidence supporting routine adjuvant therapy, patient selection is fundamental. Adjuvant radiation may be considered for patients with positive surgical margins, unresectable localized disease, or recurrent disease not manageable with complete resection. Systemic therapies and medical management are most appropriate for patients with unresectable, metastatic, or biochemically active disease in patients where surgical cure is no longer feasible. Across all scenarios, treatment decisions should be individualized and guided by multidisciplinary consensus and existing expert guidelines [7, 77, 80, 75, 91].

## 17. Surgical Outcomes

### 17.1. Recurrence Rates by Surgical Approach

Parathyroid carcinoma is associated with a high risk of localized recurrence following surgical treatment, though reported rates vary widely across series. In the most extensive retrospective review to date of 234 patients, Wang et al. reported recurrence rates approaching 30–50%, with the majority of recurrences occurring within the first 2–5 years after initial surgery [66]. Similarly, the NEKAR international multicenter study confirmed that recurrence is common despite apparently complete resections, highlighting the unpredictable biologic behavior of parathyroid carcinoma [10]. Contemporary reviews and institutional series have reported recurrence rates as high as 50–60%, particularly in cases managed with limited parathyroidectomy or when capsular disruption occurs at the index operation [3, 7, 9, 90, 92]. These findings underline the central role of surgical techniques in long-term disease control.

### 17.2. Disease-Free Survival and Overall Survival

Long-term outcomes in parathyroid carcinoma demonstrate substantial variability. Across institutional and population-based studies, 10-year disease-free survival has been reported to range from 49% to 79%, while overall survival ranges from approximately 49% to 87% [4, 16, 17, 66]. In a large single-institution series spanning 43 years, Harari et al. reported a median overall survival of 14.3 years (range, 10.5–25.7 years) [13]. Most notably, tumor burden alone does not drive mortality, but complications of persistent or recurrent hyperparathyroidism also contribute, including renal failure, cardiovascular disease, and metabolic derangements [3, 18].

### 17.3. Impact of Adequacy of Initial Surgery

The completeness of the initial operation represents the most important modifiable prognostic factor in PC. Multiple studies show that the first surgery offers the best opportunity for durable disease control, as subsequent reoperations are rarely curative and are associated with cumulative morbidity [7, 10, 90, 92]. Incomplete resections, capsular disruption, or positive margins significantly increase the risk of recurrence and reduce disease-free survival [3, 7, 9]. Notably, population-level data suggest that more extensive surgery does not consistently improve overall survival compared with parathyroidectomy alone, highlighting that surgical extent alone is not a marker for oncologic adequacy [59]. Achieving complete tumor excision without capsular violation is fundamental for localized control.

### 17.4. Institutional Volume Effects

Institutional experience markedly impacts outcomes in PC. In the same high-volume institutional series, Harari et al. demonstrated that patients whose initial operations were performed at a specialized endocrine surgery center experienced improved overall survival (P = 0.037) and significantly lower complication rates (P < 0.001) compared with those treated at outside institutions [92]. These findings are supported by systematic reviews and interdisciplinary management models that emphasize early referral to specialized centers, where appropriate surgical coordination and adherence to oncologic principles may reduce tumor spillage and improve long-term outcomes [8, 75].

### 17.5. Biochemical Outcomes

Postoperative biochemical remission, defined by normalization of serum calcium and parathyroid hormone (PTH) levels, is a favorable prognostic indicator in PC. In the NEKAR multicenter study, achieving biochemical remission after initial surgery was independently associated with improved outcomes, with an odds ratio of 0.023 for adverse events [12]. Conversely, persistent or recurrent hypercalcemia is strongly associated with increased morbidity and mortality, frequently caused by renal, cardiovascular, and metabolic complications as opposed to tumor progression [18, 20, 21, 75].

### 17.6. Reoperation Burden

Recurrent disease frequently necessitates repeated surgical intervention, being a major factor in cumulative morbidity and diminished quality of life. In the long-term institutional experience reported by Harari et al., patients underwent an average of three neck reoperations for recurrent disease, with a reported range of 1–11 procedures over the disease course [92]. Each successive operation increases surgical complexity and the risk of recurrent laryngeal nerve injury, hypoparathyroidism, and scar-related complications, stressing the importance of achieving definitive disease control at the initial operation [3, 7].

### 17.7. Margin Status and localized Control

Surgical margin status is a key determinant of progression-free survival in patients with localized disease. Positive or close margins are consistently associated with higher rates of local recurrence and reduced disease-free survival across institutional series and systematic reviews [3, 7, 9, 90, 92]. Margin involvement reflects either advanced local invasion or inadequate surgical technique and compromises long-term oncologic control. Achieving a complete tumor removal with negative margins at the initial operation, even if requiring *en bloc* resection of adjacent thyroid or soft tissue, is fundamental for durable disease control.

### 17.8. Outcomes in Metastatic Disease

Metastatic parathyroid carcinoma carries a markedly worse prognosis. In a pooled systematic review of published cases, the median overall survival following diagnosis of metastatic disease was 36 months [22]. Surgical resection of metastatic lesions, when feasible, was independently associated with improved overall survival, with a hazard ratio of 0.48 compared with nonsurgical management [22]. Bone metastases were linked to a significantly worse prognosis, whereas lung metastases were more responsive to surgical control. Despite multimodal therapy, management in this setting remains mainly palliative, with surgery primarily aimed at biochemical control and symptom relief.

### 17.9. Prognostic Factors and Risk Stratification

Although parathyroid carcinoma lacks a universally accepted staging system, several clinicopathologic and molecular prognostic factors have been consistently identified. Advanced T stage, nodal involvement, distant metastases, positive surgical margins, and failure to achieve postoperative biochemical remission are associated with worse outcomes [3, 7, 8, 15, 90]. Immunohistochemical markers, including elevated Ki-67 proliferative index (≥5–10%) and loss of parafibromin expression, have emerged as independent predictors of recurrence, metastatic potential, and reduced disease-free survival, refining risk stratification beyond traditional anatomic parameters [7, 8].

### 17.10. Time to Recurrence and Surveillance Implications

Recurrence most commonly occurs within the first several years following initial treatment. Median time to recurrence has been reported at approximately 27–33 months in institutional series, with the majority of recurrences occurring within 2–5 years [3, 66]. However, late recurrences beyond a decade have been documented, supporting the necessity of long-term, and often lifelong, surveillance [90, 92, 93]. Follow-up strategies should incorporate serial biochemical monitoring and periodic imaging to aid in early detection of recurrent or metastatic disease.

## 18. Complications

Parathyroid carcinoma is associated with substantial perioperative and long-term morbidity, reflecting both the aggressive surgical management often required and the widespread effect of prolonged, severe hyperparathyroidism. Complication profiles differ meaningfully from those observed in benign primary hyperparathyroidism (pHPT) and must be interpreted in the context of frequent reoperations, advanced disease at presentation, and prolonged patient survival despite recurrent or persistent disease [10, 18, 92].

### 18.1. Hypocalcemia and Hungry Bone Syndrome

Postoperative hypocalcemia is a common complication following resection of functional PC and may be severe or prolonged. In contrast to benign pHPT, patients with PC frequently present with longstanding and severe hypercalcemia accompanied by advanced skeletal demineralization, predisposing them to hungry bone syndrome after tumor removal. This syndrome is characterized by rapid skeletal uptake of calcium and phosphate, resulting in extreme hypocalcemia that frequently requires aggressive calcium and calcitriol supplementation and extended inpatient monitoring [1, 3, 75]. Repeated cervical exploration and extensive *en bloc* resections further increase the risk of permanent hypoparathyroidism, a complication that is uncommon after initial surgery for benign disease but contributes substantially to long-term morbidity in PC [10, 90, 93, 94].

### 18.2. Recurrent Laryngeal Nerve Injury and Vocal Cord Paralysis

Recurrent laryngeal nerve (RLN) injury represents one of the most consequential surgical complications in PC. Tumor adherence to or invasion of the RLN is not uncommon, and oncologic resection may necessitate nerve sacrifice to achieve local disease control [59, 95]. Several institutional series have reported both unilateral and bilateral vocal cord paralysis following surgery for PC, with bilateral paralysis carrying considerable consequences for airway protection, phonation, and quality of life, and in some cases necessitating tracheostomy [5, 93]. These outcomes contrast sharply with benign pHPT surgery, in which permanent RLN injury is rare and typically limited to isolated cases [79].

### 18.3. External Branch of the Superior Laryngeal Nerve Injury

Injury to the external branch of the superior laryngeal nerve (EBSLN) is frequently underrecognized but may result in clinically significant voice dysfunction, especially impacting pitch modulation and vocal endurance. This complication is especially relevant in PC, where wide dissection and reoperative surgery are common. Within institutional endocrine surgery series, the use of intraoperative neuromonitoring has been associated with a lower incidence of EBSLN injury compared with visual identification alone, consistent with broader neuromonitoring literature [1, 93].

### 18.4. Complications of Hypercalcemia

Unlike many solid malignancies, mortality in PC is more commonly related to effects of uncontrolled hypercalcemia as opposed to direct tumor burden. Chronic hypercalcemia contributes to progressive renal dysfunction, including nephrocalcinosis and renal failure, which may ultimately require dialysis, as well as cardiac arrhythmias, neuropsychiatric manifestations, and severe skeletal disease, including fractures and osteitis fibrosa cystica [8, 10, 54, 90]. These systemic complications may persist or recur despite surgical intervention, particularly in patients with recurrent or metastatic disease, and often drive long-term clinical management.

### 18.5. Reoperation-specific Complications

Reoperation is frequently required in PC due to high rates of persistent or recurrent disease. Complication rates are consistently higher in re-operative parathyroid surgery compared with initial operations, reflecting scar tissue, distorted anatomy, and compromised nerve visualization. Multiple studies demonstrate increased risks of recurrent laryngeal nerve injury and permanent hypoparathyroidism with repeat cervical exploration, with cumulative morbidity rising with each subsequent operation [5, 10, 59, 90, 93]. This stepwise increase in surgical risk represents a defining challenge in the long-term management of PC.

### 18.6. Parathyromatosis

Parathyromatosis is a rare but serious complication resulting from capsular disruption or tumor seeding, most associated with fine needle aspiration or intraoperative tumor rupture. This condition leads to multiple foci of hyperfunctioning parathyroid tissue that are difficult to fully remove surgically and may result in refractory hypercalcemia [54,95]. Its occurrence is largely preventable by avoiding preoperative biopsy and by precise surgical technique emphasizing en bloc resection without capsular violation.

### 18.7. Quality-of-Life Considerations

Quality-of-life (QOL) impairment in PC extends beyond traditional surgical morbidity. Many patients experience prolonged survival defined by repeated operations, chronic hypocalcemia requiring lifelong calcium and vitamin D therapy, and lasting voice dysfunction following RLN or EBSLN injury. Severe hypocalcemia and permanent hypoparathyroidism are associated with neuromuscular symptoms, neuropsychiatric effects, renal complications, and sustained reductions in patient-reported quality of life [25]. In contrast, benign pHPT is characterized by low long-term morbidity, with permanent hypoparathyroidism occurring in fewer than 2–3% of patients at high-volume centers and parathyroidectomy consistently associated with symptomatic and quality-of-life improvement [10]. Together, these distinctions highlight PC as a unique clinical entity characterized by extended survival and high cumulative surgical and metabolic morbidity [5, 10, 18, 59, 90].

### 18.8. Prognostic Factors

The key prognostic factor in parathyroid carcinoma is margin status at initial surgery [87]. Multiple cohort, multicenter, and database studies show that patients with R0 resection (microscopically negative margins) have greater survival rates than those with positive margins [55]. Negative margins also correlate with lower recurrence and decreased need for further operations [87]. Margin status is mainly determined by the initial surgical approach. Later operations generally cannot compensate for failure to achieve a negative margin during the first surgery. Capsular disruption and incomplete excision are more common when patients undergo limited resection. In contrast, an en bloc resection increases the likelihood of achieving negative margins by removing the tumor and surrounding tissue [83]. Capsular rupture during surgery or tumor adherence to surrounding structures are major contributors to margin positivity and recurrence. This allows tumor cells to disseminate locally, which is why a precise and meticulous surgical technique is essential. Incomplete resection is strongly associated with early recurrence and the need for reoperation [12, 87].

### 18.9. Extent of Resection

The extent of surgical resection is an important factor in parathyroid carcinoma, though its independent prognostic value is still debated [83]. Although en bloc resection has long been standard, recent studies indicate that more extensive surgery does not always improve survival, particularly in the absence of evident invasion [96]. Instead, resection quality outweighs mere extent. Outcomes depend primarily on attaining complete tumor removal during the initial operation [66, 83].

## 19. Lymph node involvement

Unlike other head and neck cancers, parathyroid carcinoma mainly spreads locally, with recurrence more frequently resulting from incomplete resection than nodal invasion [87]. Lymph node metastases are rare but may signal more advanced disease. Nodal involvement likely reflects tumor biology rather than serving as an independent predictor of survival. Studies indicate that although nodal disease may be associated with increased recurrence, it does not dependably affect overall survival [66]. Current evidence does not support routine prophylactic lymph node dissection. Therefore, a selective approach should be used, reserving dissection for clinically or intraoperatively suspicious lymph nodes [66, 96].

## 20. Genetic Markers

The CDC73 (HRPT2) tumor suppressor gene is the most well-established genetic abnormality in parathyroid carcinoma [97]. Loss of function of this gene leads to reduced parafibromin expression, a protein involved in cell cycle regulation and associated with more aggressive tumor behavior [53, 67]. Testing for CDC73 mutations may help distinguish malignant from benign parathyroid disease [53]. However, its role in clinical practice remains mainly prognostic and does not significantly influence surgical decision-making currently [97, 98].

## 21. Summary of Included Studies

Across the included studies, several consistent patterns emerge. Recurrence is common in parathyroid carcinoma, with reported rates ranging from approximately 30% to 60%, depending on the study [66]. This variation is largely related to the effectiveness of the initial surgery. Patients who undergo complete tumor removal with negative margins at the first operation consistently have better outcomes and lower recurrence rates [66, 87]. In contrast, patients who require reoperation for persistent or recurrent disease tend to have worse outcomes and are less likely to achieve long-term disease control [12]. Some studies also suggest that the timing of recurrence is important. Patients with early recurrence tend to have worse outcomes compared to those with delayed recurrence, which suggests that tumor biology plays a role in disease progression [67]. Despite these consistent findings, variability across studies persists due to differences in surgical approach, patient populations, and reporting methods. However, the overall trend remains clear that outcomes are largely dependent on the success of the initial surgical management [66].

Evidence indicates that parathyroid carcinoma behaves primarily as a locally aggressive disease rather than as one that spreads early [87]. Most recurrences are driven by incomplete resection instead of early metastatic spread. This again highlights how important the initial surgery is in determining outcomes. There is also variability in outcomes across studies, which may be explained by differences in tumor biology, including molecular factors and timing of recurrence. Studies show that early recurrence is associated with worse outcomes [67].

## 22. Discussion

### 22.1. Consensus vs Controversy

There is strong agreement across the literature that achieving negative margins during the initial operation is the most important factor in determining outcomes [87]. This point is consistent in most studies. However, controversy persists over how aggressive the resection should be and whether a more extensive resection is always necessary [97].

### 22.2. Comparison across Surgical Strategies

Traditionally, *en bloc* resection has been considered the preferred approach, especially when there is suspicion of invasion. In these cases, removing the tumor and surrounding tissue increases the likelihood of achieving a negative margin [83]. However, more recent studies suggest that a more aggressive approach does not always lead to better outcomes [96]. Some patients who undergo limited resection still do well, if the tumor is completely removed and the capsule is not disrupted. On the other hand, patients with incomplete resection tend to have higher recurrence rates regardless of the type of surgery performed [66,87]. This shows that the quality of the resection is more important than the extent alone.

### 22.3. Clinical Implications

From a clinical standpoint, the main goal should always be complete tumor removal during the initial operation [87]. The surgical approach should be based on intraoperative findings rather than applying the same aggressive strategy to every patient. Routine lymph node dissection is not supported by the data and should only be performed when there is clear evidence of nodal involvement [66, 96]. In addition, patients need close, long-term follow-up, as recurrence can occur years after the initial surgery [67].

### 22.4. Limitations of Existing Evidence

One of the main limitations in the literature is the rarity of parathyroid carcinoma. Because of this, most studies are retrospective and involve small sample sizes, which makes it difficult to draw strong conclusions or directly compare results across studies [66]. There is also variability in how outcomes are reported, including differences in definitions of recurrence and survival. In addition, surgical techniques and management strategies have changed over time, which may affect reported outcomes [97].

## 23. Future Directions

Future research should focus on larger studies to improve the quality of evidence. Standardizing outcome reporting would also help improve comparisons across studies. There is also growing interest in molecular markers, which may help predict outcomes more accurately and guide future management [97, 98].

## Conclusion

Parathyroid carcinoma is a rare disease, but outcomes are largely determined by complete tumor removal during the initial surgery [87]. The most important factor is achieving a negative margin, as this is consistently associated with lower recurrence and better long-term outcomes [55]. Although en bloc resection has traditionally been recommended, evidence suggests that the extent of surgery is less important than the quality of the resection^3^. What matters most is complete tumor removal without capsular disruption.

Patients with incomplete resection are more likely to develop recurrence and often require additional operations, which are less effective [12]. Lymph node involvement does not appear to play a major role in survival, and routine lymph node dissection is not supported by current data [96]. Instead, it should be performed selectively when there is clear evidence of nodal disease. Overall, parathyroid carcinoma behaves more like a locally aggressive, recurrent disease rather than one that spreads early [87]. This again highlights how critical the first operation is.

## Figures and Tables

**Table 1:** Clinical and Biochemical Features Suggestive of Parathyroid Carcinoma.

Feature	Clinical Significance	References
Serum calcium >14 mg/dL	Highly suggestive of malignancy	[7], [10]
PTH >3–10 times upper limit of normal	Strong predictor of carcinoma	[7], [15]
Palpable neck mass	Rare in benign disease	[7]
Vocal cord paralysis	Suggests local invasion	[7], [18]
Severe skeletal disease	Indicates aggressive disease	[7], [16]
Renal insufficiency or nephrolithiasis	Associated with malignancy	[7], [10]
Imaging evidence of invasion	Supports malignant diagnosis	[6], [9]

## Key Points

- Parathyroid Carcinoma is a rare and aggressive malignancy that accounts for less than 1% of primary hyperparathyroidism.
- Immunohistochemical markers such as loss of parafibromin and elevated Ki-67 are under review and helpful in evaluating the presence of parathyroid carcinoma.
- Preoperative differentiation between parathyroid carcinoma and adenoma remains difficult due to overlapping clinical and imaging features.
- Definitive diagnosis of parathyroid carcinoma requires histopathological evidence of capsular, vascular, or perineural invasion.
- The initial surgical operation is the most critical intervention for improving outcomes.
- Complete en bloc resection at the initial operation with negative margins is the most important modifiable factor in improving long-term outcomes.
- Disruption of the capsule and incomplete en bloc resection are associated with higher rates of locoregional recurrence.
- Routine lymph node resection lacks evidence of benefit and is not recommended in the absence of clinically suspicious nodes.
- Morbidity and mortality in parathyroid carcinoma are primarily driven by uncontrolled hypercalcemia rather than tumor burden.
- Lifelong biochemical surveillance with serial calcium and PTH monitoring is essential due to the risk of delayed recurrence.
